# Prevalence of Burnout among Primary Health Care Staff and Its Predictors: A Study in Iran

**DOI:** 10.3390/ijerph16122249

**Published:** 2019-06-25

**Authors:** Ehsan Zarei, Fariba Ahmadi, Muhammad Safdar Sial, Jinsoo Hwang, Phung Anh Thu, Sardar Muhammad Usman

**Affiliations:** 1 Department of Health Services Management, School of Management and Medical Education, Shahid Beheshti University of Medical Sciences, Tehran, Iran; zarei_1980@yahoo.com; 2 Deputy of Health Affairs, Kermanshah University of Medical Sciences, Kermanshah, Iran; afariba18318@yahoo.com; 3 Department of Management Sciences, COMSATS University Islamabad (CUI), Islamabad 44000, Pakistan; safdarsial@comsats.edu.pk (M.S.S.); susman@comsats.edu.pk (S.M.U.); 4 The College of Hospitality and Tourism Management, Sejong University, 98 Gunja-Dong, Gwanjin-Gu, Seoul 143-747, Korea; 5 Faculty of Finance and Accounting, Nguyen Tat Thanh University, Ho Chi Minh City 700000, Vietnam

**Keywords:** burnout, primary health care, occupational health, Iran

## Abstract

Burnout, which is an emerging challenge in health systems, is very common among primary health care (PHC) workers. The aim of this study was to investigate the level of burnout among PHC workers, and its predictive factors, in a region in the west of Iran. In this cross-sectional study, all the health network staff (*n* = 539) were enrolled. The data collection instrument was the Maslach Burnout Inventory (MBI), which consists of 22 items and the three subscales of emotional exhaustion (EE), depersonalization (DP), and personal achievement (PA). High scores in EE and DP and low scores in PA are indicative of high burnout. Logistic regression was used to determine the predictors of high burnout. The data were analyzed using SPSS version 16. The findings showed that 90.5% of the staff had high DP, 55.3% had high EE, and 98.9% had low PA scores. Also, 52.9% (277 people) of the staff suffered from high burnout. Single people (OR = 3.33), less experienced employees (OR = 9.09), people aged over 35 years (OR = 2.35), physicians (OR = 1.72), and staff with permanent employment (OR = 5.0) were more likely to suffer high levels of burnout. We conclude that burnout is a common problem in PHC workers. Less experienced, younger, single employees and physicians were more at risk of suffering from high burnout. Preventive measures, such as strengthening social skills, communication competencies, and coping strategies, and reduction of risk factors such as job stress, are suggested for reducing employees’ risk of burnout.

## 1. Introduction

Primary health care (PHC) includes providing services for disease prevention, treatment, management and rehabilitation. It is an essential part of the health system in achieving its goal of improving the health of populations [1,2]. A PHC system needs a competent, effective, and motivated workforce to provide high-quality services to meet the stated goal. Notwithstanding this, structural reforms in the health system have led to heavy workloads, which may lead to employees burnout [3]. Health worker’ burnout is a considerable challenge for health systems, with potential negative consequences for the health care provider, the patients and the health care organizations [4,5,6,7,8,9]. For this reason, burnout has attracted considerable research attention in the past two decades, in both developing and developed countries [10,11].

Occupational burnout is a psychological state that was first defined by Maslach in the 1980s as the end state of chronic stress associated with work [12]. This condition is composed of emotional exhaustion (EE), depersonalization (DP), and reduced personal achievement (PA). EE refers to the reduction or loss of emotional resources and the feeling of being emotionally exhausted due to work, along with the feeling that there is nothing to offer psychologically to others. DP is negative attitudes and feelings and a lack of sensitivity and empathy towards the people who are being served. Reduced PA is the tendency to assess oneself and one’s work negatively, with the avoidance of interpersonal relationships, low productivity, and lack of resistance to stress [13].

Burnout is common among employees who work in stressful environments [4]. Based on the extensive body of evidence available, burnout syndrome is very common in care professions such as healthcare workers [3,7]. Health professionals work in emotional situations and face the physical, economic, social, and psychological problems of patients and their families [14]. They experience a lot of stress due to the need for quick responses to the needs of patients and their families, the high workload, and long hours of work in a complex working environment [13]. Exposure to these conditions can lead to burnout [11]. Recent studies indicate a high level of stress and burnout in healthcare workers. Studies from around the world, including on physicians [6,15,16], nurses [10,17,18], physiotherapists [14,15,16,17,18,19], primary health care workers [20,21,22] and other health professionals [4,7,13,23], have reported prevalence rates of burnout from 2.6% to 75%. In addition, the results of two studies from Iran showed that the prevalence of burnout in PHC workers is 17.3% to 34.5% [21,24].

Burnout has serious effects on the physical and mental health of health care providers. Cardiovascular disorders, musculoskeletal disorders, depression, and anxiety are all associated with burnout [13,25]. It is also related to absenteeism, low productivity, reduced job satisfaction, and the intention to leave a job. Also, burnout has negative effects on care quality and patient satisfaction [7,8,16,21,25,26]. In addition, health system costs increase due to high turnover, absenteeism, reduced staff motivation, poor quality of care, and possible medical errors [5,8,10,13,16,20]. Burnout syndrome is the result of the complex interaction of social and individual factors. Job stress, workload, role conflict, organizational changes, and reduced social and organizational support are factors that affect the onset of burnout [13,25]. Also, some demographic variables such as age, gender, marital status, educational level, and years of professional experience are assumed to be associated with burnout [26,27,28].

The study of work issues among health sector employees in Iran has been more focused on hospital staff (especially physicians and nurses) and community health workers have been neglected. Few studies have been performed on burnout among PHC workers in Iran [24,25,26,27,28,29]. PHC workers perform several tasks and are under great pressure, especially in rural and deprived areas. On the other hand, they have limited career development and opportunity for job promotion [21,30]. In addition, Iran’s Health Transformation Plan (HTP) has been under way since 2014. The HTP includes reforms to primary health care, including a non-communicable diseases control plan and implementation of an electronic health record system. This has led to new tasks (in addition to primary tasks) and more expectations of primary health care workers [31], and due to inadequate numbers of primary healthcare workers [30], it has led to an increased workload and stress for them [32]. In the study by Peikan pour et al., job burnout among health staff was identified as one of the weaknesses of the HTP due to the increase in the number of patient visits [33]. On the other hand, in terms of improvements in pay, primary care workers have not been considered, and the focus has been on hospital staff, especially physicians, which has resulted in feelings of inequity and discrimination among community health workers [32,33,34].

Together, these factors can create the potential for burnout. Therefore, identifying the prevalence of burnout and its predictive factors can help health administrators to develop appropriate preventive interventions. The aim of this study was to examine the level of burnout among PHC workers and its predictive factors in a region in the west of Iran.

## 2. Materials and Methods 

### 2.1. Design and Sampling 

This cross-sectional study was conducted in 2018 in a western region of Iran. Based on the structure of the health system in Iran, each county (district) has a health network that provides health services at the primary and secondary levels. Each health network consists of a general district hospital and a set of rural health houses, urban health posts, and urban and rural health centers. Health houses are staffed with community health workers (called “Behvarz”, with two years of training undertaken in specialized training centers) and other facilities are staffed with health professionals including: physicians; nurses; midwives; laboratory, radiology and dentistry technicians; and experts on disease control, environmental health, occupational health, mental health, school health, family health and health education [2]. The surveyed health network included 30 rural health houses, five urban health posts, four urban and rural health centers, and one general hospital, which provide services to 71,000 residents. All the health network staff with clinical roles in health services delivery (*n* = 539) were enrolled in the study, and consisted of 267 primary-level and 272 hospital staff. Having at least one year of work experience in the health network was the inclusion criterion of our study.

### 2.2. Instrument and Procedure

The data collection instrument was the Maslach Burnout Inventory (MBI), which is the gold-standard of burnout surveys in the field of medicine [35]. The first part of the questionnaire includes items on demographic variables, and the second part consists of 22 items in the three subscales of emotional exhaustion (EE), depersonalization (DP), and personal achievement (PA). This questionnaire has been used in various studies in Iran to measure burnout, and its Persian translation is available [21]. The Cronbach’s alpha coefficient of this questionnaire was calculated at 0.82, indicating the acceptable internal consistency of the tool.

This questionnaire is rated based on a 7-point Likert scale, ranging from 0 (never) to 6 (every day). Due to the multi-dimensionality of the burnout structure, the scores of each subscale are reported separately and cannot be added up as a total score [36]. According to Maslach, high scores in EE and DP and low scores in PA are considered as high burnout [7,37]. Burnout level is classified into three groups, low, moderate, and high. The cut-off points for this classification [23,37] are presented in Table 1. Questionnaires were distributed in person at the workplace of the staff. After describing the purpose of the study and assuring the confidentiality of information, staff consent to participate in the study was obtained and they were given a week to complete the questionnaire. After a week, completed questionnaires were collected. In total, 524 completed questionnaires we returned (response rate = 97%).

### 2.3. Data Analysis 

The data were analyzed using SPSS. In the descriptive section, frequency, mean, and standard deviation were used, A *t*-test was also run to compare the mean scores of the burnout subscales in terms of demographic variables. Logistic regression was used to determine the predictors of high burnout with *p*-values less than 0.05 considered statistically significant.

### 2.4. Ethical Considerstions 

This study was approved by the Ethics Committee of Shahid Beheshti University of Medical Sciences (code of ethics: IR.SBMU.PHNS.REC.1397.132). The employees voluntarily participated in the study and their oral consent was obtained before filling in the questionnaire.

## 3. Results

According to the findings, 43% of the staff were male and 63.4% had an academic degree. The mean age of the staff was 33.5 ± 8.3 years, and the work experience mean was 9 years, which ranged from 1 to 28 years. Also, 12.6% of the employees were physicians and 60.7%worked on temporary contracts. In addition, most of the staff (68.5%) worked in PHC centers (Table 2).

Table 3 shows the status of burnout. Based on the findings, 90.5% of staff had high DP, 55.3% had high EE, and 98.9% had low PA. According to the classification, 52.9% (*n* = 277 people) had high burnout (high EE and DP along with low PA). 

Table 4 demonstrates the status of burnout scores in terms of the demographic variables of the employees. Findings showed that, except for the work setting, in all the demographic variables— which included gender, age, work experience, profession, type of employment, education level, and marital status—the difference in the mean score of EE was significant (*p* < 0.05). EE was significantly higher among physicians, female participants, employees under the age of 35 years, employees with fewer than 10 years of work experience, employees with permanent employment, and academic degree holders. The findings showed that the mean score for DP was significantly different based on age, work setting, and marital status (*p* < 0.05). The mean score of DP was higher in single employees, those under the age of 35 years, and employees working in hospitals. The mean score of PA was significantly different based on age, work experience, profession, type of employment, work setting, education level, and marital status (*p* < 0.05). The mean score of PA was lower in employees under the age of 35 years, those with less than 10 years of work experience, those with academic degrees, non-physician staff, temporary employees, and staff working in health centers.

Logistic regression was used to determine the predictors of high burnout. An omnibus test was used to evaluate the regression model, which showed that the regression model had a suitable goodness of fit (*p* < 0.05). Our findings showed that five demographic variables, which included age, work experience, profession, type of employment, and marital status, were predictors of high burnout (*p* < 0.05). In other words, employees over the age of 35 years (OR = 2.35), single individuals (OR = 3.33), employees with less work experience (OR = 9.09), physicians (OR = 1.72), and employees with permanent employment (OR = 5.0) were more likely to experience high levels of burnout (Table 5).

## 4. Discussion

The aim of this study was to investigate the prevalence of burnout among PHC workers and its predictors. The findings showed that the prevalence of burnout was very high (52.9%). The prevalence of high burnout has been reported at 17.3% in Iran’s PHC system [21], 2.6% in health professionals of Ecuador [13], 7% in Brazil’s PHC staff [20], and 54% in Iranian nurses [18]. In addition, findings of the review studies showed the prevalence of burnout in medical residents to be 35.7% [16], and among physicians it was 67% [6]. One possible reason for these differences in the prevalence rates is the differences in socio-economic status of the regions and participants. Also, differences in patient expectations can affect prevalence rates; as patients’ expectations increase, the levels of stress and ultimately, burnout rise [13]. Organizational (e.g., organizational climate, management/leadership styles, and communication) and personal (e.g., demographic characteristics, individual attitudes, and personality) factors in health systems can also contribute to these differences [5,10,38]. In addition, the classification of the prevalence rates and the cut-off points for high levels of burnout were very different among various studies [37].

The percentage of high EE was higher in our study compared to others. This figure was 35.7% in the Iranian PHC staff [21], 17.2% in Ecuadorian health professionals [13], 43% in Brazilian PHC staff [20], 64% in Palestinian hospital emergency staff [4], and 32.7% in Malawian health workers [23]. Also, the results of review studies showed the prevalence of EE was 20%–81% among Arab health workers [5], 31% in nurses [17], 38.9% in medical residents [16], and 72% in physicians [6]. The EE subscale mainly reflects aspects of the organizational environment such as role conflict, role ambiguity, participation in decision making, the reward system, and social support networks [35]. Therefore, employees who show a high degree of EE feel they cannot participate in decisions or lack social support and are generally not compatible with their organizational climate. The findings of a study among nurses reflected that a limited role in care planning had a positive effect on EE [39]. EE is associated with mental health problems and leads to anxiety and depression and ultimately, lower efficiency [4,7].

The prevalence of DP was very high in our study; this figure has been reported as 49.6% in Iran [21], 13.5% in Ecuador [13], 17% in Brazil [20], 38% in Palestine [4], 22.8% in Malawi [23], and 20%–80% in Arab countries. The findings of previous review studies indicate that the rate of high DP was 24% among nurses, 43.6% in medical residents, and 68% in physicians. DP represents the interpersonal dimension of burnout. The feeling of apathy towards patients due to too much contact with them and lack of adequate support from supervisors and colleagues can be the main reasons for DP. Findings of a study performed in Iran showed that leadership, lack of support, and poor relations with physicians had a positive effect on DP among nurses [39].

Based on the findings, we found that almost all employees experienced reduced PA. The rate of reduced PA in studies conducted in Iran [21,24,29], Ecuador [13], Brazil [20], Palestine [4], and Malawi [23] ranged from 8.8% to 34.6%. In review studies, reduced PA was reported in 13%–86% of Arab health workers [5], 38% of nurses [17], 34.3% of medical residents [16], and 63.2% of physicians [6]. In general, PA scores in studies from the medical field have been significantly lower [17]. The feeling of reduced PA leads to reduced motivation, poor performance, and ultimately, reduced job satisfaction. Many PHC workers in Iran who work in rural health houses have no opportunities for promotion in their careers, leading to the feeling of lacking considerable occupational achievements.

Burnout was higher among young employees. The findings of previous studies confirm this point [11,20,27,40]. Maslach stated that this syndrome is more influential in young professionals who work in high-risk and client-centered fields such as health services [19], and as age increases burnout decreases. One possible reason may be the uncertain horizons and the lack of job security among young employees. In addition, older employees, over time, learn how to manage occupational stress and adapt and become resistant to burnout [4]

Single employees were exposed to about three times greater risk of burnout than married employees. In general, being single is an important risk factor for burnout [19,40]. Unmarried workers have less social and family support in professional issues [35]. Previous studies have suggested that family and social support have an inhibitory effect on burnout [17].

A high degree of burnout was more prevalent among less experienced employees. In previous studies, a similar finding was reported [17,20]. Experienced employees may feel more confident about their duties [13]. Also, having longer working experience is associated with a more advanced career and higher job satisfaction. Our findings also showed that personal achievements of experienced employees were greater.

Burnout among physicians was higher than in other professionals, which is in line with the findings of previous studies [5]. The prevalence of burnout among physicians was almost twice as high as that of general population in the United States [8]. A review study in France showed that the prevalence of burnout was 49% among physicians [15]. Studies conducted in recent years have reported that the rate of burnout among physicians was higher than 50% [26]. This difference seems to be due to the great burden of responsibilities in the medical profession and the fact that these professionals have to make more critical decisions under conditions of uncertainty [13].

Although gender was not a predictor of high burnout, a higher percentage of female employees suffered from high burnout and emotional exhaustion. Perhaps this is because women often have a dual role, as both a health care provider and a mother, which can cause stress and depletion of overall energy [9].

This study had some limitations. Due to the cross-sectional nature of the study, strong causal relationships cannot be inferred. Future studies using longitudinal designs can help better understand the relationships between variables. Also, this study, like all others using self-report data, may be associated with social desirability bias and participants might have expressed their opinions too strongly or weakly.

## 5. Conclusions

This study showed that burnout is a common problem among PHC workers. Overall, 52.9% of participants reported high levels of burnout. Less experienced, younger, and single employees and physicians were more at risk for high burnout. Further studies are needed to investigate this issue to develop effective interventions to reduce the level of burnout among employees. For employees at risk of burnout, preventive measures, such as strengthening social skills, communication competencies, coping strategies, and risk reduction factors are suggested. Improving job satisfaction through rewards, incentives, career development, and educational opportunities can lead to an increase in the sense of personal achievement. Depersonalization can be reduced through employee involvement, role resolution, and support from supervisors and colleagues.

## Figures and Tables

**Table 1:** Classification of burnout level.

Level	Low	Moderate	High
EE (9 items, 0–54)	0–18	19–26	27–54
DP (5 items, 0–30)	0–5	6–9	10–30
AP (8 items, 0–48)	39–48	32–38	0–31

Note: emotional exhaustion (EE), depersonalization (DP), and personal achievement (PA).

**Table 2:** Characteristics of the sample (*N* = 524).

Variable		N	%
Gender	Male	225	42.9
	Female	299	57.1
Age	35≤	338	64.5
	<35	186	35.5
Education	Primary	192	36.6
	Academic	332	63.4
Marital status	Single	189	36.1
	Married	335	63.9
Profession	Physician	66	12.6
	Non-physician	458	87.4
Work setting	Health centers	359	68.5
	Hospital	165	31.5
Work experience	10≤	375	71.6
	<10	149	28.4
Employment type	Permanent	206	39.3
	Temporary	318	60.7

**Table 3:** Scores on the MBI subscales.

MBI Subscale	Mean ± SD	Low		Moderate		High	
		N	%	N	%	N	%
EE (0–54)	29.68 ± 8.2	13	2.5	221	42.2	290	55.3
DP (0–30)	16.89 ± 4.8	0	0	50	9.5	474	90.5
PA (0–48)	20.92 ± 5.1	518	98.9	6	1.1	0	0

**Table 4:** Comparison of the mean burnout scores in terms of demographic variables.

Variable		EE	DP	PA
Gender	Male	27.3	17.1	21.0
	Female	31.5	16.7	20.8
	P	0.001	0.4	0.7
Age	35≤	30.5	17.2	19.9
	<35	28.2	16.3	22.7
	P	0.003	0.04	0.001
Education	Primary	26.1	16.9	22.1
	University degree	31.7	16.9	20.2
	P	0.001	0.9	0.001
Marital status	Single	33.5	17.5	20.2
	Married	27.5	16.5	21.3
	P	0.001	0.03	0.04
Profession	Physician	35.8	16.6	24.0
	Non-physician	27.9	15.1	20.5
	P	0.001	0.15	0.003
Work setting	Health centers	29.2	16.2	20.1
	Hospital	30.6	18.4	22.8
	P	0.11	0.001	0.001
Work experience	10≤	31.4	16.9	20.4
	<10	25.2	16.7	22.2
	P	0.001	0.5	0.001
Employment type	Permanent	30.8	16.8	22.0
	Temporary	28.9	16.9	20.3
	P	0.007	0.7	0.001

**Table 5:** Logistic regression: Predictors of high burnout.

Demographic Variables		High Burnout		OR	P
		N	%		
Gender	Female	177	59.2	1.58	0.06
	Male	106	47.1	ref	
Age	35≤	215	63.4	2.56	0.001
	<35	68	36.5	ref	
Education	University degree	224	67.4	2.04	0.07
	Primary	59	30.7	ref	
Marital status	Single	136	72.3	3.33	0.001
	Married	145	43.4	ref	
Profession	Physician	59	89.3	1.72	0.002
	Non-physician	224	48.9	ref	
Work setting	Hospital	88	53.3	1.24	0.38
	Health centers	195	54.3	ref	
Work experience	10≤	255	68.1	9.09	0.001
	<10	28	18.7	ref	
Employment type	Permanent	132	64.1	5.0	0.001
	Temporary	148	47.1	ref	
